# Nucleic Acid Hybrids as Advanced Antibacterial Nanocarriers

**DOI:** 10.3390/pharmaceutics12070643

**Published:** 2020-07-08

**Authors:** Sybil Obuobi, Nataša Škalko-Basnet

**Affiliations:** Drug Transport and Delivery Research Group, Department of Pharmacy, Faculty of Health Sciences, UiT The Arctic University of Norway, Universitetsveien 57, 9037 Tromsø, Norway; natasa.skalko-basnet@uit.no

**Keywords:** nucleic acid nanocarriers, hybrids, bacterial infections, antimicrobial resistance, DNA nanostructures

## Abstract

Conventional antibiotic therapy is often challenged by poor drug penetration/accumulation at infection sites and poses a significant burden to public health. Effective strategies to enhance the therapeutic efficacy of our existing arsenal include the use of nanoparticulate delivery platforms to improve drug targeting and minimize adverse effects. However, these nanocarriers are often challenged by poor loading efficiency, rapid release and inefficient targeting. Nucleic acid hybrid nanocarriers are nucleic acid nanosystems complexed or functionalized with organic or inorganic materials. Despite their immense potential in antimicrobial therapy, they are seldom utilized against pathogenic bacteria. With the emergence of antimicrobial resistance and the associated complex interplay of factors involved in antibiotic resistance, nucleic acid hybrids represent a unique opportunity to deliver antimicrobials against resistant pathogens and to target specific genes that control virulence or resistance. This review provides an unbiased overview on fabricating strategies for nucleic acid hybrids and addresses the challenges of pristine oligonucleotide nanocarriers. We report recent applications to enhance pathogen targeting, binding and control drug release. As multifunctional next-generational antimicrobials, the challenges and prospect of these nanocarriers are included.

## 1. Introduction

**Figure 1:**
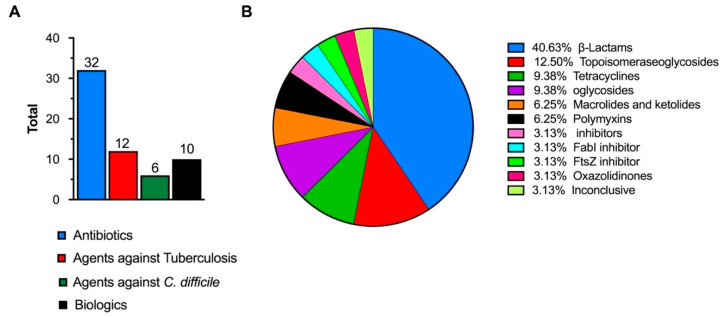
(**A**) Total number of antimicrobial agents in clinical development [13]. (**B**) Percentages of specific antibiotic classes in clinical development (inconclusive refers to agents classified as having inconclusive data or no agreement among the advisory group) [13]. The image is drawn using data from [13].

Deadlocked in the evolutionary arms of microorganisms, the competitive medical world struggles to contain the spread of multi-resistant infections, due to the ineffectiveness of conventional antimicrobials [1]. Many of these pathogens result in prolonged illnesses that cause 700,000 estimated deaths each year [2,3]. In Europe alone, recent estimates indicate that more than 670,000 infections in the European Union/ European Economic Area Countries (EU/EEA) are due to antibiotic resistance, with approximately 33,000 deaths annually [4]. As a direct consequence of these infections, EUR 1.5 billion related costs are accrued by healthcare systems in EU/EEA countries [5]. One of the major driving forces behind the acquisition and spread of AMR is the indiscriminate use of antimicrobial agents [6,7]. This exerts ecological pressure on bacteria and contributes to the emergence and selection of multidrug resistance genes which are easily transmitted between humans and animals [8]. Among the commonly isolated strains, the ESKAPE pathogens (*Enterococcus faecium, Staphylococcus aureus, Klebsiella pneumoniae, Acinetobacter baumannii, Pseudomonas aeruginosa* and *Enterobacter spp*.) constitute a panel of highly recalcitrant bacteria that have garnered immense interest, given their ability to evade common antimicrobial therapies [9,10]. These pathogens have been identified among the WHO’s priority list drawn up in the bid to fortify research efforts and prioritize new drug molecules that outwit multidrug resistance mechanisms [11,12]. However, of the sixty antibiotics and biologics under clinical development (Figure 1), limited benefit over existing treatments has been identified, and very few of them target the most critical gram-negative pathogens [13,14]. With a weak antibiotic pipeline caused by the long development timeline of pre-clinical candidates, declining private investment and the lack of innovation in developing new antimicrobial therapies, new approaches are warranted to effectively combat drug resistant infections [15]. 

Engineered nanocarriers represent a growing area of interest for the delivery of antimicrobial cargos [16]. These platforms have been shown to address the inherent toxicity, high dosage requirements (for intracellular infections and resistant pathogens), rapid in vivo degradation and short half-lives (e.g., peptide-based therapeutics) of antimicrobials [17]. Efficient delivery of drugs to the infection site at a controllable frequency and dosage have also been demonstrated [18,19]. Additionally, given the role of commensals in maintaining homeostasis and the effect of sub-lethal antibiotics on the microbiome, these nanocarriers are a promising solution that can enhance pathogen targeting [20,21]. A myriad of lipid based (e.g., liposomes, micelles), metallic (e.g., gold nanoparticles) and polymeric nanocarriers (e.g., nanogels) have been developed as delivery systems for antimicrobial drugs [22,23,24,25]. While lipid-based carriers such as liposomes comprise the most well-known and widely investigated platform, they are challenged by vesicle instability, low entrapment efficiency and sterilization difficulties [16]. Moreover, conventional liposomal vesicles undergo rapid elimination from the blood stream and have poor cell specificity [26]. Additionally, overcoming the bacteria cell envelope is a major obstacle for liposomal formulations and for non-fusogenic liposomes, local extracellular release [27] can compound toxic effects. On the other hand, metallic nanoparticles are limited by toxicity concerns (e.g., naked iron oxide nanoparticles) and aggregation issues (e.g., silver nanoparticles) [28,29]. Similarly, the chemical composition of polymeric nanoparticles, physicochemical properties (e.g., surface potential) and/or their subsequent degradation products have also been associated with cell toxicity, stress and inflammatory responses [30,31,32]. Recent strategies to uncover advanced delivery systems with improved physicochemical properties and antibacterial effects include the development of nucleic acid nanocarriers and their conjugates. These nanocarriers possess excellent biocompatibility, biodegradability and targeting properties [18]. For instance, aptamer-based systems have been shown to be efficient as promising tools against bacterial biofilms, possess excellent antimicrobial activities, inhibit immune cell invasion and reduce the effect of bacterial toxins [33]. 

Within this context and as an emerging field, deoxyribonucleic acid nanotechnology (e.g., DNA origami technique) exploits the programmability of single-stranded DNA molecules to generate simple or complex self-assembled nanostructures of variable shapes, well-defined forms and precision. Early studies on the applications of DNA nanostructures mainly focused on applications in cancer therapeutics. Overall, the prevailing rationale for using nanocarriers in cancer therapeutics, is the crucial requirement for an ideal nanocarriers that will overcome systemic toxicity of chemotherapeutics to improve drug inefficacy and biodistribution [34]. Additionally, it has also become imperative to develop systems that bypass drug resistance mechanisms attributable to chemotherapy failure [35]. In addressing these challenges, DNA nanocarriers have shown superiority given their high drug loading and efficient cellular uptake [36]. For instance, the high loading capacity of DNA nanocarriers for doxorubicin (a primary model anticancer drug) is easily achieved at room temperature, and relies on its ability to strongly intercalate with dsDNA [37]. Moreover, remarkable molecular mechanisms that drive reconfiguration of the nanostructures in response to various stimuli (e.g., membrane receptors, cellular peptides and restriction enzymes)—by relying on the incorporating of DNA aptamers, enzyme-specific DNA sequences and hybridization chain reactions during fabrication—have been demonstrated [38]. These features have consequently enabled researchers design DNA nanocarriers that can sense, adapt or react to the tumor microenvironment, accumulate within tumors and control the release of chemotherapeutics. Converse to the success observed with cancer therapy, the exploitation of DNA nanostructures in antibacterial therapy has not been widely reported, potentially due to the challenges posed by the bacteria cell envelope and its smaller size. Excitingly, some recent reports have demonstrated the transferability of some of these strategies to ensure bacterial targeting and to impart antibacterial potency via the exploitation of DNA nanostructured conjugates by leveraging non-covalent or covalent interactions. It is evident from these reports that there is an immense potential to revolutionize antibacterial therapeutics by exploiting DNA nanostructured hybrids as advanced antimicrobial platforms. We define DNA hybrids as nanostructured DNA carriers that are complexed or conjugated with organic and inorganic molecules. This review will hence focus on discussing DNA nanostructures, and report an up-to-date overview of DNA nanostructured conjugates fabricated with organic and inorganic materials, before embarking on describing how these nanocarriers have been applied in bacterial therapy. To conclude, we will discuss their challenges as antibacterial therapeutics and their exciting prospect. 

## 2. DNA Nanocarriers

### 2.1. Principles for Fabrication 

DNA is a double-stranded molecule that carries genetic information in all living systems. Following the initial proposal of immobile Holliday junctions by Seeman and subsequent generation of DNA scaffolds, the development trajectory of DNA nanotechnology has seen several breakthroughs that have made a profound impact on the design of unique nanostructures [39,40]. The central mechanism for designing DNA nanostructures relies on the complementarity of the base pairs to direct cohesion of single-stranded DNA, and is based on the Watson-Crick hydrogen bonds. The versatility, homogeneity, controllable size and shape, biocompatibility and ease of functionalization identify these platforms as excellent nanocarriers [41]. Within DNA nanotechnology, there are generally two design paradigms for fabricating nanostructures and their crosslinked scaffolds. These are the DNA origami approach and the DNA tile approach. In the former technique, complex nanostructures can easily be formed in a fast and robust way that generates high yield nanostructures. It utilizes a long single strand of DNA (M13 bacteriophage DNA), which is folded with short staple strands [42]. On the other hand, using the tile approach, short synthetic strands of DNA are assembled together into larger nanostructures [43]. Although the tile approach is characterized by greater expense and low yields [18], the fabrication process is simpler and uses less sequences. Using these two approaches, numerous well-defined two-dimensional (2D) and three-dimensional (3D) nanostructures have been generated with promising applications in biosensing, bioimaging, diagnosis and drug delivery [18]. Despite the abovementioned advantages and prospect of these nanostructures, pristine DNA nanostructures face a myriad of obstacles that can limit their clinical translation.

### 2.2. From Test Tube to Biological Applications: Current Challenges

Although structural DNA nanotechnology enables the generation of DNA nanostructures with rather precise control of size, geometry and presentation of ligands, the structures need to be compatible with biological environments to survive the biological pathway to their targets [44]. Many DNA nanostructures have poor stability within physiological environments. Thus, to assure their clinical translation, their behavior within the cellular environment and biological system is essential [45]. Numerous authors have tested the performance of DNA nanostructures using in vitro models/conditions, suggesting that the findings can be translated into in vivo fate. For instance, Walsh and colleagues reported unaided uptake of a FRET-labeled DNA tetrahedron via receptor-mediated endocytosis [46]. Previous work by Rosi and colleagues, however, suggested that the 3D configuration of DNA nanostructures aids their cellular delivery, in comparison with naked DNA strands [47]. However, Lacroix et al. proposed that nuclease digestion of dye-labeled DNA nanostructures might result in the release of free dyes into solution, leading to false evidence of nanostructure uptake [48]. Understandably, this will mean that the encouraging findings in the cellular conditions will fail to translate in biologically relevant applications. These discrepancies often involve the use of non-standardized methods to evaluate the factors affecting the cellular fate of DNA nanostructures, and remain one of the main obstacles to their translation. For instance, very often the cellular uptake is followed by DNA nanostructures attached to cyanine dyes; it is important to highlight that these dyes can direct the uptake of DNA strands themselves [49]. Very recently, Green and colleagues critically summarized the factors that need to be standardized, to assist in easier translation from laboratory conditions into clinic [45]. In another effort, Kim and colleagues developed tumor-specific drug carriers by screening a library of self-assembled nucleic acid cages in tumor-bearing mice [50]. Interestingly, the authors concluded that tumor specificity was closely related with serum stability, cancer cell uptake efficiency and macrophage evasion rate, and suggested that the library-based strategy can be an efficient way to develop anti-cancer nanomedicines with tumor specificity and enhanced potency.

In recent years, other promising approaches that are easy to implement, cost-effective and could significantly strengthen the translation of many already existing and newly designed therapeutic and diagnostic structures have been investigated. For example, Anastassacos and co-workers proposed an easy, effective and scalable chemical cross-linking approach to develop stable DNA nanostructures [51]. Using a chemical cross-linking reagent glutaraldehyde that strongly binds distinct PEGylated oligolysines on the surface of DNA nanostructures, additional knots were tied into the electrostatic net. This intervention led to improved stability in the presence of nucleases extending beyond 300 hours. The 400-fold increase in stability observed with PEGylated oligolysine alone was elevated by another 250-fold. Seeking to ensure successful applications of DNA nanostructures as bacterial therapeutics, this study exemplifies how modifications of pristine DNA nanostructures can enhance stability in the biological environment with the prospect of promoting intended delivery to targeted pathogens. This enhanced stability, coupled with targeted delivery, would thus lead to improved bioavailability and reduced side effects associated with therapeutic antimicrobial cargos. 

## 3. Nucleic Acid Hybrid Nanocarriers 

To ensure the competitiveness of DNA nanostructures over existing nanocarriers, conjugation with organic and inorganic materials (Table 1) has been proposed to improve stability and specificity, attenuate immune response or enable delivery via other routes. An unprecedented array of hybrid structures can be fabricated using the modularity of DNA nanostructures, wherein several functional features are incorporated in a facile fashion. Intuitively, a simpler approach that has also been utilized to tackle the abovementioned challenges is to simply coat the nanostructures (non-covalently) using lipids, proteins (e.g., bovine serum albumin) or other synthetic polymers (e.g., polyethylene glycol) [52,53]. Nevertheless, the fusion of organic and inorganic materials with DNA nanostructures is an essential first step to fabricate multifunctional nanocarriers that can be applied in antimicrobial therapy. This section provides an overview of hybrid systems developed using this approach. 

### 3.1. Fabrication with Organic Materials

The fabrication of nucleic acid amphiphiles increases the hydrophobicity of DNA and is one of the most common strategies used to enhance cellular uptake of therapeutic nucleic acids, as well as an opportunity to deliver both hydrophobic and hydrophilic drugs. DNA amphiphiles have been used to self-assemble one- (1-D), two- (2-D) and three-dimensional (3-D) nanostructures, wherein the hydrophobic portions can comprise synthetic polymers, fluorescent dyes or lipids [54,55]. These conjugates are widely applied in biosensing, diagnostics, nanoreactors and in drug delivery, with further functionalization shown to enhance targeting and biocompatibility [56,57]. Among the diverse hydrophobic ligands that can be conjugated to oligonucleotides, cholesterol, tocopherol, porphyrin, peptides, steroid and single chain fatty acid modifications have been reported [58,59,60]. Cholesterol functionalization remains one of the most popular ways to anchor nucleic acid sequences with hydrophobic groups. These amphiphiles have the capacity to self-assemble into predictable morphologies (e.g., spherical micelles and vesicles) due to their tendency to microphase separate; these effects are dependent on the number of hydrophobic ligands, the type of residue and its position [61,62,63]. 

A critical challenge of amphiphilic DNA molecules is their tendency to aggregate. This often eliminates structural control, which is the primary advantage of DNA nanotechnology. Addressing the issue of aggregation with cholesterol modification, Ohmann and coworkers recently showed that aggregation of ssDNA was sequence-dependent, whereas, in the self-assembled constructs, the position of the cholesterol modification was the dominant factor [63]. More importantly, the authors outlined the significant influence of particular bases (AAA bases had no aggregation) preceding the cholesterol modification and overhangs (six nucleotide overhangs on the complementary sequence) in suppressing aggregation.

Advancements in DNA nanotechnologies have facilitated the arrangement of proteins as functional molecular components in 3D configurations with nanometer precision, thereby combining the diverse functionality of proteins with the programmability of DNA for nanocarrier fabrication [80,81]. These hybrid systems can be extended to assemble multiple proteins, construct enzyme cascades and fabricate biosensors or biochips [82,83]. The generation of nucleic acid-protein conjugates is a promising and versatile tool to enhance gene delivery, modify the solubility, biological activity and stability of therapeutic cargos or the nanostructure [84,85,86,87,88]. As an unrivaled class of macromolecular drugs with high specificity, proteins are biodegradable, easily metabolized and amenable to surface modifications [89]. In fact, many highly functional nanomachines in nature are built from protein macromolecules [90]. The natural amphiphilicity of the protein biopolymer is ideal, as it encourages good interaction with both solvents and drug molecules (hydrophilic and hydrophobic) [88]. These DNA-protein conjugate systems can be achieved via non-covalent (e.g., biotin-streptavidin, nickel-nitrilotriacetic acid-hexahistidine and antibody-hapten interactions) or covalent strategies (e.g., disulfide or maleimide coupling and biorthogonal chemistry) using proteins such as streptavidin, antibodies, zinc finger proteins and engineered proteins containing His-, Snap- and Halo-tags [81,90]. In designing nucleic acid-protein hybrid nanocarriers, incorporating recognized multifunctional features such as stimuli responsiveness (to both nucleases and proteases), excellent biocompatibility and integration of targeting moieties (to drive cellular specificity) is highly attractive for anticancer [67] and antimicrobial therapeutics. 

**Figure 2:**
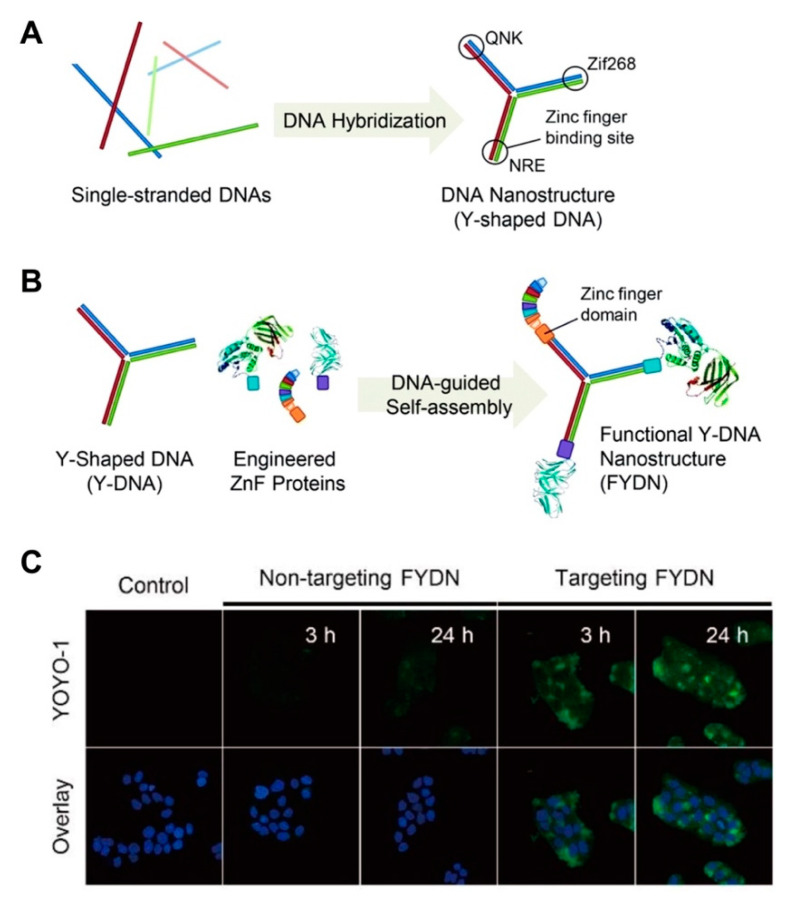
(**A**) Construction of a functional nucleic acid-protein hybrid system using Y-shaped monomer having three different zinc finger binding sites. Reproduced with permission from [93]. Copyright Royal Society of Chemistry, 2012. (**B**) Schematic diagram of site-specific conjugation of the zinc finger fused proteins with the Y-DNA monomer for the construction of the targeted hybrid. Reproduced with permission from [93]. Copyright Royal Society of Chemistry, 2012. (**C**) Confocal images of the cellular uptake of the protein-bound hybrid system in lung adenocarcinoma (HCC827) cells compared to the bare monomer. Green dye indicates YOYO-1 dye proteins bound to the hybrid. DAPI dye was used to stain the nuclei. Reproduced with permission from [93]. Copyright Royal Society of Chemistry, 2012.

Despite the immense potential of DNA-protein hybrid systems, conventional techniques are often challenged by poor site-specificity, which produces heterogeneous product mixtures and can render proteins dysfunctional or cause nonspecific binding to cells; an obvious challenge to translational medicine [80,91,92]. Seeking to address this limitation, Ryu and co-workers recently reported a modular design to assemble functional DNA nanostructures [93]. The authors designed oligonucleotides with zinc finger binding sites encoded within each oligomer (Figure 2A,B). Following spontaneous hybridization, the Y-shaped self-assembled nanostructure yielded homogeneous zinc-fused proteins with different biological roles (i.e., targeting moiety, a molecular probe and a therapeutic cargo). The hybrid system showed strong resistance to exonuclease activity (7.5% digestion) compared to naked DNA (87.4% degradation) after 6 h. This consequently resulted in the efficient cytosolic delivery of a tumor suppressor protein in cancer cells (Figure 2C).

### 3.2. Fabrication with Inorganic Materials

DNA nanostructures have also been explored for the spatial organization of discrete inorganic nanoparticles such as gold, silver and quantum dots (Table 1) [44]. Among the vast metals used in fabricating DNA hybrids, gold nanoparticles are among the most well-developed and reported in imaging, drug delivery, nanofabrication, sensing and plasmonic rulers [94,95]. Although there are a lot of nuances in these hybrids, the nature of the sequences (appropriate thymine rich sequence), adequate sonication, placement of a spacer between the thiol moiety and the nucleobases, as well as an appropriate salt aging influences the number of DNA units that can be successfully tethered [96]. Nucleic acid gold nanoparticulate hybrids can be used in the fabrication of spherical nucleic acids. These nanocarriers are known to possess minimal toxicity, require facile and well-established synthesis, display remarkable stability against degrading enzymes and achieve high cellular uptake [97]. One significant challenge of spherical nucleic acids is the low uptake of ultra-small nanoparticles, which significantly reduces therapeutic efficacy [98]. Additionally, efficient clearance remains an essential pre-requisite to ensure safe translation into clinical practice [99]. Recent efforts to address this include DNA-mediated self-assembly of gold-DNA nanostructures designed by Huo and co-workers [73]. The authors utilized near-infrared (NIR) radiation to liberate ultrasmall gold nanoparticles of 2 nm sizes. By further modifying the gold nanoparticles with an oncogene silencing sequences (i.e., *c-myc*), enhanced nucleus penetration and transfection efficiency were observed. This transformable feature of the hybrids is attractive and provides an excellent model for the design of hybrid nanovehicles with transformative antimicrobial properties. 

**Figure 3:**
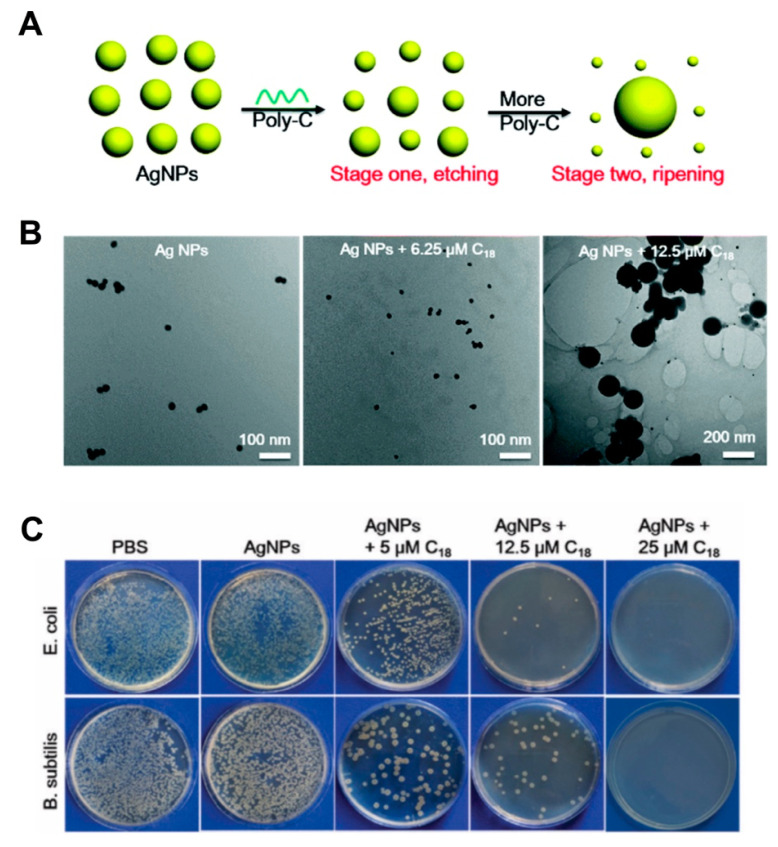
(**A**) Schematic representation of etching and ripening stages of silver nanoparticles in the presence of poly-C DNA sequences. Reproduced with permission from [77]. Copyright Royal Society of Chemistry, 2019. (**B**) TEM images of the silver nanoparticles mixed with different concentrations of C18 DNA. Reproduced with permission from [77]. Copyright Royal Society of Chemistry, 2019. (**C**) Photographs of plated *E. coli* and *B. subtilis* after different treatments with the hybrid. Reproduced with permission from [77]. Copyright Royal Society of Chemistry, 2019.

The use of silver has been widely documented since the 20th century for the control of infections [100]. Similarly, transition metals of the d-block, metals and metalloids from group 13 to 16 of the periodic table have been considered as antimicrobial agents [101]. Given their low propensity to develop resistance these metals are attractive alternatives to address the challenge of antimicrobial resistance. In fact, the strong efficacy of metals as antibiofilm agents and their potency against persister cells that are impervious to antibiotics have been reported [102,103,104,105,106]. Over the last decade, there has been widespread use of silver ions, and their efficacy has been widely documented as commercial products (e.g., wound dressings) and medical device coatings (e.g., urinary catheters) [107,108,109]. Among the many metal-based nanosystems reported in literature, silver nanoparticles are largely recognized for their excellent broad-spectrum bactericidal actions, high efficacy at low doses and low propensity to drug resistance (compared to antibiotics) [110,111,112]. Additionally, silver nanoparticles are electrochemically and catalytically active and display a size-dependent surface plasmon resonance. Despite their poorer stability in comparison to gold nanoparticles (due to their tendency to oxidize and form irreversible aggregates in high salt concentrations), the sharp plasmon resonances and larger extinction coefficients of silver nanoparticles are especially attractive for molecular detection applications [113,114]. Efforts to address the above-mentioned challenges of silver by fabricating hybrids with DNA nanostructures with well-ordered structures have been reported. However, the therapeutic application of silver nanoparticles is often limited by the physicochemical properties (e.g., size) and their aggregation-prone nature [29,115,116]. One strategy to address this was reported by Hu and coworkers via poly-cytosine DNA induced etching of silver nanoparticles [77]. Etching/dissolution of the silver nanoparticles was seen at low concentrations of the DNA whereas with increasing DNA, ripening was shown (Figure 3A,B). In addition to showing small nanoparticles, the authors indicated that in presence of salt ions, a red shift of the plasmonic peak was observed, which is contrary to the expected decrease seen during silver aggregation. Enhanced cytotoxicity against cancer cells and enhanced antibacterial activity against both Gram-positive and Gram-negative bacterial cells was also shown (Figure 3C). Comparatively, using the silver nanoparticles alone resulted in low antibacterial activity due to the size, whereas the Poly-C (C_18_) DNA showed no antibacterial activity. 

There has been rather less research progress into DNA-directed self-assembly of quantum dots, due to high ionic concentrations and relatively high temperatures required for DNA nanostructure fabrication. Pre-requisites necessary to facilitate the DNA-directed self-assembly of quantum dots include a high chemical and photonic stability, strong binding affinity with DNA oligonucleotides, high colloidal stability over a wide range of buffer conditions, high quantum fluorescence efficiency and a high spectral tunability [78]. Such stable conjugates that withstand precipitation at high temperatures and ionic strengths have been reported by Deng et al. [78]. Greater stability of their hybrids stems from embedding the DNA within the shell by taking advantage of chimeric phosphorothiolated phosphorodiester ssDNA (ps-po-ssDNA), which are directly inserted within the thick shell (cadmium selenide/zinc sulphide) during its synthesis over the core. The authors reported chemical, photonic and colloidal stability of the conjugates. In another study, Zhang and colleagues described a one-step synthesis route for the fabrication of self-assembled quantum dot DNA hydrogels under physiological conditions [79]. Featuring a phosphorothioate backbone, spacer and a DNA binding domain, the authors leveraged the high binding affinity between quantum dots and the phosphorothioate backbone to immobilize the quantum dots within the scaffold. The hybrid system showed a 9-fold increase in doxorubicin potency, and was efficient in vivo in xenografted breast cancer tumors.

## 4. Nucleic Acid Hybrid Nanocarriers as Advanced Systems against Bacterial Infections

Conjugating nucleic acid sequences with organic and inorganic materials has proven to be a feasible strategy to fabricate hybrid systems that address many of the imminent challenges of chemotherapeutics. Like chemotherapy, antibiotic use is associated with low permeability across cellular membranes, low activity within bacteria cells and systemic toxicities that preclude sufficient bactericidal activity [117,118]. This is attributable to the bacteria cell wall, which is four to five times thicker in Gram-positive bacteria than in Gram-negative bacteria [119], as well as multiple efflux pumps that reduce net permeability of antibiotics into the bacteria cells [120]. Further changes in the transportation of antibiotics through the bacteria cell wall can cause drug resistance [121]. A current innovative approach to address this is to facilitate cellular accumulation via efficient uptake and to prevent degradation [117]. However, in persisting bacteria, most antimicrobial agents have no effect since many of the bacterial cells are dormant [122]. Moreover, genetic mutations in resistant bacteria also influence genes encoding drug targets, enzymes and regulators of transporters highlighting the need for new antimicrobial therapies [122]. With a complex interplay of factors driving resistance mechanisms in nature and an increasingly large number of multidrug-resistant infections, nanosystems that can be adapted to combat disease-causing bacterial pathogens using other mechanisms of action from traditional antibiotics, as well as delivering antimicrobial therapies, are warranted. With the diverse attractive features of nucleic acid hybrids in cancer therapy, it is not inconceivable that further optimization of the hybrids can improve bacteria targeting and bactericidal activity using antibiotic-loaded and antibiotic free systems. Although rather unexploited to-date, some recent reports have already made advances in this field and demonstrated the feasibility of utilizing nucleic acid nanocarriers (both tile-based nanostructures and DNA origami nanostructures) and their hybrids against planktonic and biofilm infections. This section reviews these reports and classifies their use into three main areas: (a) nucleic acid hybrids with enhanced cellular uptake; (b) nucleic acid hybrids to control or sustain delivery of antimicrobials; (c) nucleic acid hybrid with enhanced binding to bacteria cells.

### 4.1. Nucleic Acid Hybrids with Enhanced Cellular Uptake

Several strategies have been investigated to increase the cellular uptake of antimicrobial therapies. Among these, the use of non-viral carriers, such as cell penetrating peptides, has received greater attention due to their minimal toxicity, low levels of immunogenicity, ease and flexibility of assembly [123]. Nevertheless, poor specificity to bacteria cells, protease sensitivity and resistance in cells linked to encoding translocating proteins such as SbmA has been reported [124,125,126]. Nucleic acid hybrid systems can be exploited as alternative vectors to promote uptake of antibacterial agents without detrimental effects. Within this context, novel theragnostic DNA nanoscaffolds were fabricated by Setyawti and colleagues for the delivery of antibiotics against Gram-positive and Gram-negative bacteria [127]. In this work, the authors utilized tetrahedral nanostructures conjugated with gold nanoclusters for bacteria detection. By leveraging the intercalation capacity of Actinomycin D to DNA, the nanocarriers ensured that the otherwise impermeant Actinomycin D would cross the bacteria cell membrane. The authors reported that the hybrid was more readily taken in by *S. aureus* (1.2-fold higher uptake than *E. coli*). Nevertheless, an uptake of at least 70% was observed within 3 h in both pathogens. The authors argued that the internalization of the nanoparticles in bacteria cells was eased by the small size and pyramidal shape of the nanostructures. Overall, the nanoscaffold enhanced the reduction of bacteria cells by 65% compared to the observed 42% achieved by the free drug. In reference to the proposed intracellular release of the bound cargo, the authors hypothesized that intracellular release of therapeutic cargo was mediated by the abundant deoxyribonucleases (DNase), which form a part of the innate capability of both *E. coli* and *S. aureus* to attain transformation. A dose-dependent degradation of the hybrid system was demonstrated in vitro when incubated with increasing concentrations of DNase I. Additionally, the conjugated red-emitting gold nanoclusters enabled trackability of the hybrid system to detect and assess the efficacy of the system. In addition to antibiotics, tetrahedral nanostructures have been shown to enhance the cellular uptake of other antimicrobial therapies, such as peptides. Liu and colleagues reported the enhanced antimicrobial activity and improved stability of antimicrobial peptides against bacteria degradation when loaded in DNA tetrahedral nanostructures [128]. Using the GL13K peptide as the model drug, the authors showed increased uptake of the peptide and enhanced stability in a protease-rich extracellular environment. At the optimal ratio, the efficacy of the hybrid system was enhanced from ~85% to ~99% against *E. coli* and from ~1% to ~32% against *P. gingivalis*.

**Figure 4:**
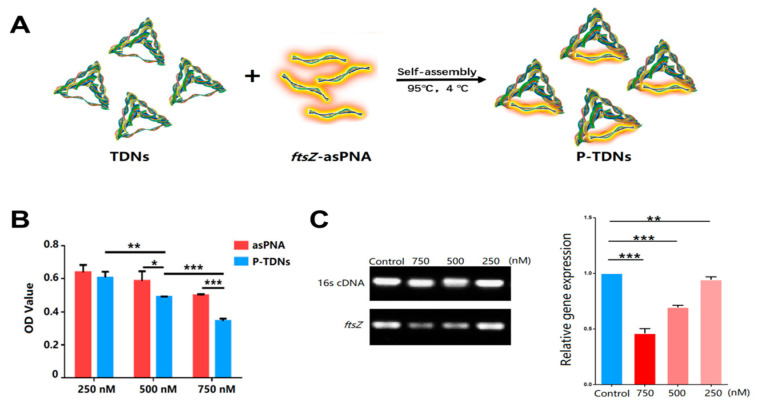
Example of a nucleic acid conjugate with inherently antimicrobial properties. (**A**) Schematic representation of the DNA tetrahedral hybrid system for delivering antisense peptide nucleic acids. Reproduced with permission from [125]. Copyright American Chemical Society, 2018. (**B**) In vitro antibacterial properties of the hybrid system against methicillin resistant *S. aureus* (MRSA) cells at varying concentrations of the antisense peptide nucleic acid alone and the hybrid system. Reproduced with permission from [125]. Copyright American Chemical Society, 2018. (**C**) Semiquantitative (agarose gel image) and quantitative (bar graph) analysis of *ftsZ* gene expression in MRSA cells after 24 h treatment at different doses, in comparison to universal primer control (16s cDNA). Reproduced with permission from [125]. Copyright American Chemical Society, 2018. * *p* < 0.05; ** *p* < 0.01; *** *p* < 0.001.

The specificity of antisense oligonucleotide therapies to their mRNA targets and subsequent translational effects contributes to their success in inhibiting a wide range of bacteria genes that drive resistance or promote bacteria growth [129]. To address the poor stability of unmodified antisense oligonucleotides, antisense peptide nucleic acids are a new generation of DNA analogues, with improved stability stemming from their resistance to enzymatic degradation [130,131]. Nevertheless, similar to their unmodified sequences, entry into bacteria cells is challenging for many. To address this limitation and extend the application of tetrahedral nanostructures, Zhang et al. developed a tetrahedral DNA nanostructure for the delivery of an antisense peptide nucleic acid against methicillin-resistant *S. aureus* infections [125]. Targeting a highly conserved protein involved in bacteria cell replication (FtsZ), specific inhibition of the *ftsZ* gene was demonstrated by the authors after applying the hybrid system (Figure 4A). The authors demonstrated a concentration-dependent reduction in *ftsZ* gene expression and bacteria cell growth following incubation with the antisense peptide nucleic acid delivery system (Figure 4B,C). The *ftsZ* gene has previously been validated by several studies as a suitable target for antibacterial interventions, thus, the hybrid systems evaluated in this study provide a background for the optimization of new anti-staphylococcal therapies with improved cellular uptake [125]. 

In another work, Readman et al. developed a non-toxic DNA tetrahedron nanoparticle for the delivery of a targeted anti-bla_CTX-M-group 1_ antisense peptide nucleic acid [129] to increase bacterial sensitivity to antibiotics. In the presence of cefotaxime, a dose-dependent potentiating efficacy was observed, wherein the hybrid system reduced the minimum inhibitory concentration (MIC) of cefotaxime from 35 to 16 mg/L against *E. coli*. This effect was, however, not achieved in the presence of the antisense peptide nucleic acid alone, demonstrating the advantage of employing the DNA nanostructure to fabricate the hybrid system. Instead of targeting single genes, Zhang et al. developed tetrahedral nucleic acid nanostructures for multi-targeted antisense oligonucleotide delivery [132]. Towards inhibiting biofilm formation and virulence, the authors reported on the reduced expression of *gtfBCD*, *gbpB* and *ftf* genes. A dose-dependent uptake of the delivery system by *S. mutans* cells after 12 h was demonstrated, with less mature biofilms formed after treatment with the antisense loaded system. Since the authors sought to inhibit genes responsible for the extracellular polysaccharides (EPS), reduced thickness of the biofilms due to disruption in the cells’ ability to synthesis EPS was shown. In addressing potential mechanisms governing uptake, post-cell penetration and dissociation of the antisense oligomer or the degradation of the tetrahedral nanostructure upon entering the bacterial cytoplasm to release the synthetic antisense component was proposed by Readman et al. [129]. However, the mechanisms governing uptake of DNA nanostructures into bacteria cells remains unknown. It has been demonstrated that Gram-negative bacteria recognize sequence-specific DNA fragments [133], which may contribute to the uptake of the hybrid systems. Nevertheless, cell penetration via unidentified pathways enabled by varying the shape and size of the structures can also contribute to the enhanced uptake of the nanostructures [129].

**Figure 5:**
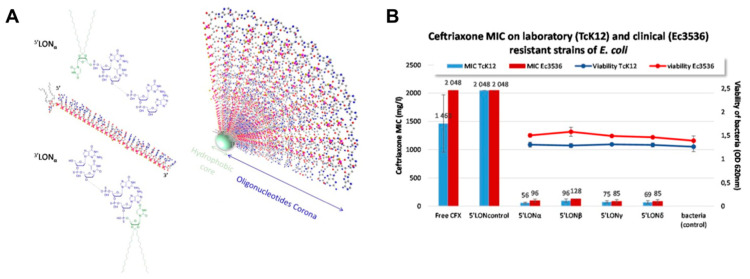
Example of a nucleic acid conjugate to restore the sensitivity of bacteria to antibiotics. (**A**) Schematic representation of the chemical structures of lipid antisense conjugates and its subsequent self-assembly to a micelle. Reproduced with permission from [134]. Copyright Springer Nature, 2020. (**B**) In vitro effect of the hybrid on the MIC of ceftriaxone and on bacteria viability of a resistant laboratory (TcK12) and clinical strain (Ec3536). Reproduced with permission from [134]. Copyright Springer Nature, 2020.

To demonstrate that other modifications of the nucleic acids can achieve a similar effect, Kauss and colleagues reported the fabrication of oligonucleotide hybrid nanostructures featuring a lipid oligonucleotide with complementary sequences to CTX-M extended spectrum -β- lactamases (ESBLs) as an efficient approach to reduce the resistance of pathogenic bacteria to antibiotic treatment [134]. The oligonucleotide conjugates self-assembled in aqueous solutions to form micelles (10 nm) with a hydrophobic lipid core and an oligonucleotide corona (Figure 5A). The most potent conjugate demonstrated a 26-fold decrease in the MIC of ceftriaxone against resistant *Escherichia coli* TcK12 (Figure 5B). It was demonstrated that the position of the lipid influenced efficacy and the authors reported that the 3´ lipid conjugates were less efficient in enhancing ceftriaxone potency. This was attributed to a more pronounced destabilization of the heteroduplex mRNA-lipid conjugated oligonucleotide, steric hinderance that avoids RNAse H cleavage of the mRNA strand or less efficient bacterial uptake. While an inhibition in lactamase activity was observed, gene and protein expression were not significantly different from the control after treatment, which was converse to the observations seen for the tetrahedral nanostructures.

### 4.2. Nucleic Acid Hybrids to Control or Sustain Delivery of Antimicrobials

**Figure 6:**
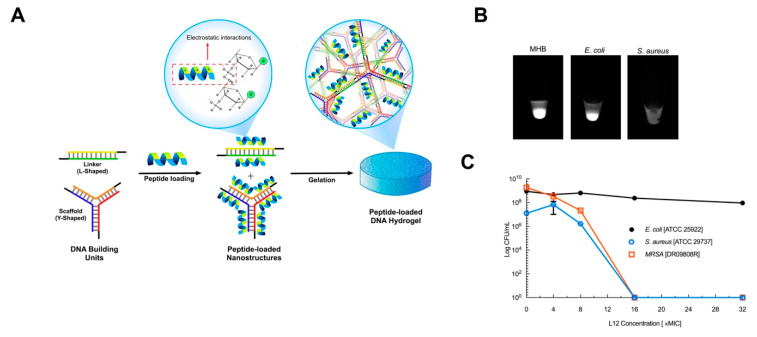
Example of nucleic acid conjugates for the delivery of antimicrobial drugs. (**A**) Schematic representation of crosslinking strategy and utility of electrostatic interaction for incorporating L12 peptide into DNA hydrogels. Image reproduced from [136]. Copyright Elsevier Ltd., 2019. (**B**) Bacteria dependent degradation of the DNA hydrogels after 24 h in MHB media, *E. coli* and *S. aureus* cultures. Image reproduced from [136]. Copyright Elsevier Ltd., 2019. (**C**) Killing efficiency of the peptide loaded gels against bacteria pathogens over 24 h. Image reproduced from [136]. Copyright Elsevier Ltd., 2019.

The use of DNA nanostructures as delivery vehicles for antimicrobial drugs has gained interest as high loading scaffolds that can control or sustain the delivery of antimicrobials. In view of tackling resistant pathogens, reported strategies have focused on the delivery of antibiotics, antimicrobial peptides and cationic microbicides. Over the last decade, there has been heightened interest in the development of antimicrobial peptide-loaded carriers to tackle resistant pathogens [135]. Nevertheless, drug delivery strategies remain at the forefront of antimicrobial peptide therapeutic development, due to the relatively poor pharmacokinetics of peptides [136]. Very few studies have evaluated the capacity of DNA based nanostructures to efficiently deliver antimicrobial peptides against infections. In our previous work, we reported for the first time the delivery of L12 antimicrobial peptide-loaded in crosslinked DNA nanostructures against topical wound infections caused by *S. aureus* (Figure 6A) [136]. 

By leveraging electrostatic interactions between the anionic DNA nanostructures and cationic antimicrobial peptides, high loading of the peptide was achieved, which, under physiological conditions, resulted in sustained release of the therapeutic peptide. To drive responsive peptide release, we relied on the presence of nucleases from pathogenic *S. aureus* and demonstrated controlled release behavior alongside superior antimicrobial activity against *S. aureus* (Figure 6B,C). For instance, in vitro killing efficiency following treatment with the delivery scaffold demonstrated rapid killing at 16× MIC for both susceptible and methicillin-resistant strains of *S. aureus*. Conversely, no efficacy was demonstrated against *E. coli* even at 16× MIC and 32× MIC drug loading, despite the similarity of the MIC values in both strains. Additionally, single application on porcine explant wound infection models resulted in potent efficacy after 24 h, alongside fast healing rates in murine excision wounds. 

In another study, crosslinked DNA nanostructures were explored as antimicrobial adhesive dressings with a green industrial microbicide [137]. The authors encapsulated tetrakis (hydroxymethyl) phosphonium sulfate (THPS), a cationic phosphonium salt via intermolecular electrostatic interactions and hydrogen bonding to achieve lasting and controllable release of the microbicide. The hydrogel system achieved complete inhibition of bacteria growth in *S. aureus* and *E. coli* models over 24 h alongside negligible toxicity over 48 h. The outstanding broad-spectrum antibacterial activity was attributed to the disruption of the bacteria cell membrane, due to the adsorption of released THPS to the negatively charged bacteria cell wall to promote subsequent lysis. Finally, in a mouse skin model, the authors demonstrated complete closure of the wound area compared to blank controls, which achieve approximately 80% wound closure after 15 days.

In another study, non-toxic lipid-modified DNA strands were equipped with different drugs by hybridization, with an aptamer for ophthalmic application. The self-assembled micellar products exhibited a lipid core and DNA corona [59]. Generally, the topical administration of ocular therapeutics requires high drug doses and frequent administration. Thus, the authors investigated essential physicochemical features paramount for achieving high bioavailability by increasing mucosal adhesion and/or association with cell membranes to enhance residence time. The number of hydrophobic bases and total number of nucleotides were shown to be the important determining parameters for efficient corneal adhesion. The hybrid system showed remarkable neomycin B and kanamycin B attachment in vivo on porcine and human corneal tissues up to 4 h, compared to the small free drug control. The kanamycin loaded nanoparticles demonstrated significant reduction in bacteria growth in bacteria keratitis model of *P. aeruginosa*. This effect was maintained over 48 h demonstrating the sustained antibacterial activity of the hybrid system. Conversely the free drug did not achieve significant growth inhibition, which stresses the pitfalls of current ocular formulations and the advantage of nucleic acid hybrid nanocarriers.

**Figure 7:**
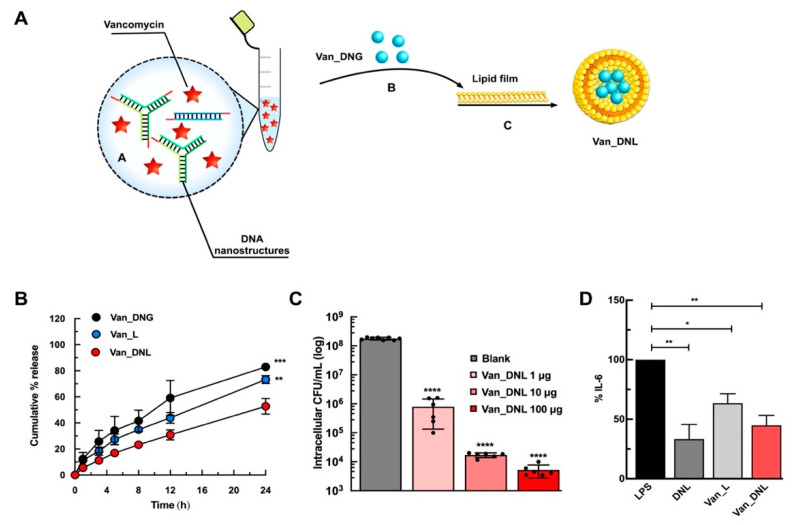
Example of nucleic acid hybrid for the delivery of antibiotics. (**A**) Schematic representation for the development of vancomycin loaded hybrids. Nucleic acid nanogels were caged together with vancomycin antibiotics within liposomal vesicles. Image reproduced from [19]. Copyright Elsevier Ltd., 2020. (**B**) In vitro sustained release of vancomycin from the hybrids (Van_DNL) compared to the liposomal (Van_L) and nanogel (Van_DNG) controls. Image reproduced from [19]. Copyright Elsevier Ltd., 2020. (**C**) Intracellular antimicrobial activity of the hybrids (Van_DNL) against macrophage infected *S. aureus*. Image reproduced from [19]. Copyright Elsevier Ltd., 2020. (**D**) In vitro anti-inflammatory activity of the hybrids (Van_DNL) after pre-treatment in lipopolysaccharide (LPS) stimulated RAW 264.7 macrophages. Reproduced with permission from [19]. Copyright Elsevier Ltd., 2020. * *p* < 0.05; ** *p* < 0.01; *** *p* < 0.001; **** *p* < 0.0001.

To demonstrate the efficacy of the hybrids in treating intracellular infections, we recently reported the fabrication of a hybrid system wherein nucleic acid nanogels were caged within liposomal vesicles for antibiotic delivery (Figure 7A) [19]. 

The central principle of the approach relies on exploiting non-covalent electrostatic interactions between cationic cargos and polyanionic DNA to immobilize vancomycin and enable precise temporal release against intracellular *S. aureus*. We proved the close proximity of the chromophores of vancomycin to the bases of the DNA nanostructures, which is attributable to aromatic stacking/hydrogen bonding/electrostatic/hydrophobic interactions. The hybrid system enhanced the loading efficiency of vancomycin and significantly sustained drug release, compared to the liposomal or nanogel controls (Figure 7B). In response to relevant enzymatic conditions to *S. aureus* infections, the hybrid system resulted in controlled release of vancomycin and DNA when treated with lipase and DNase enzymes, respectively. We achieved a concentration dependent reduction in intracellular bacteria after 24 h using the hybrid system (Figure 7C). Finally, a strong synergistic anti-inflammatory activity following endotoxin stimulation (Figure 7D) demonstrated the immense advantage of these systems as a universal platform against persistent infections.

Jeon et al. also developed a DNA-based antibiotic carrier as a vehicle for the efficient loading of a minor-groove binding antibiotic, netropsin [138]. Using rolling circle amplification, the DNA particles were rationally designed with exposed minor grooves for antibiotic binding. Surface modification via simply coating the surface of the nanocarrier with calcium was performed to control the release kinetics of the bound antibiotic. The hybrid system achieved 80% loading efficiency of netropsin within 5 min. Although the antimicrobial activity of the hybrid system was not tested, the authors demonstrated a significantly slower release of netropsin from the calcium coated hybrids, compared to the uncoated nanocarriers in over 6 days, highlighting a novel approach to modulate electrostatically bound antibiotics to nucleic acid nanocarriers.

In addition to the abovementioned non-covalent loading approaches, Kumari and colleagues developed a hybrid hydrogel system using cytosine rich ssDNA covalently attached to carbon dot (CD) and protoporphyrin IX (PpIX) [139]. The CD conjugate acted as a crosslinker and energy donor to excite the antimicrobial photosensitizer. Hydrogel formation was induced via i-motif formation which was dependent on slow reduction of the pH (from pH ~8 to ~7) in the system. The authors observed sustained release of PpIX from the hybrid system for more than a week, alongside the subsequent generation of reactive oxygen species over ten days. Finally, the expediated killing of *S. aureus* bacteria via PpIX excitation was demonstrated and attributed to energy transfer from CD or direct irradiation of PpIX with visible light, highlighting the application of hybrid nanocarriers with inherent antimicrobial activities.

### 4.3. Nucleic Acid Hybrid with Enhanced Binding to Bacteria Cells

In addition to achieving enhanced cellular uptake of antimicrobial agents, specific binding of DNA nanostructured hybrid systems with high affinity and selectivity to other bacteria targets, proteins or small molecules can be considered to improve clinical efficacy/outcome [33]. Within this context, aptamers which are small single-stranded DNA sequences have been evaluated with success as treatment modalities against bacteria biofilms and toxins [33]. For instance, considering the impact of motility on the initial attachment of bacteria to form biofilms, pretreatment of *Salmonella Choleraesuis* aptamers initiated specific binding to the flagella to prevent the formation of mature biofilms [140]. It was also demonstrated that aptamer pre-treatment substantially decreased the amount of ampicillin required to inhibit bacteria growth and thus eliminate the need for high doses of potent antibiotics. The prevention of alpha-toxin and enterotoxin B induced overexpression of cytokines via the use of aptamers have been demonstrated in *S. aureus* therapy [141]. Given the susceptibility of aptamers to enzymatic degradation by nucleases, delivery modalities and chemical modifications can improve their half-lives and stability in vivo. 

**Figure 8:**
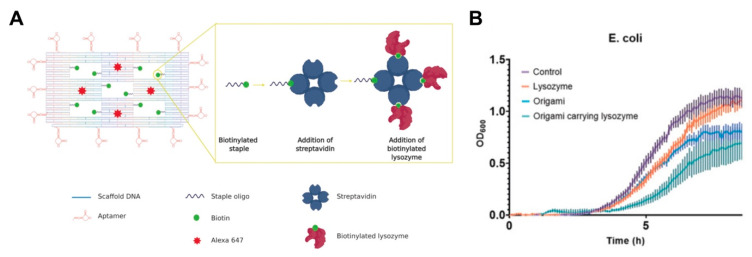
(**A**) Schematic representation of DNA origami nanostructures featuring an aptamer, lysozyme and Alexa 647 fluorescence molecules. Image reproduced from [142]. Copyright John Wiley and Sons, 2020. (**B**) Average growth curves of *E. coli* in the presence of the lysozyme alone, origami alone and the lysozyme loaded origami nanostructures. Image reproduced from [142]. Copyright John Wiley and Sons, 2020.

The use of aptamer functionalized DNA origami nanostructures for the delivery of antibacterial lysozyme was recently reported by Mela and colleagues [142]. Biotinylated staples were hybridized with DNA origami nanostructures consisting of a frame with five ‘wells’. The attachment of streptavidin to the biotin ligands enabled the loading of the biotinylated lysozymes as molecular payloads (Figure 8A). The hybrid system was functionalized with aptamers that target *E. coli* and *B. subtilis*. This approach was crucial, considering that, unlike nanostructures that have enhanced uptake in bacteria cells, these targeted hybrid nanostructures will not be endocytosed, making their access to the surface crucial. Thus, the incorporation of the aptamers around the perimeter of the origami nanostructures increased the binding ability of the system. The hybrid systems displayed fourteen aptamers at the four sides of the origami, to ensure effective aptamer-driven binding of the origami to the bacterial targets. The selected aptamers were 40 bases long and able to bind five Gram-positive and Gram-negative bacteria strains, including *E. coli* and *B. subtilis*, using sequential whole-cell selection. Conversely, the non-functionalized nanostructures exhibited minimal binding (less than 2%). It was suggested that the precise nature of the interaction between the aptamer-derivatized nanostructures and the bacterial surface directly influence the effects of the nanostructures on bacterial surface, thus inhibiting crucial bacterial functions. Additionally, in vitro growth assays demonstrated slowed bacteria growth, which was more pronounced than with the free lysozyme (Figure 8B), and highlights the capacity of origami hybrid systems to also achieve effective antibacterial effects.

## 5. Outlook

Conjugating organic and inorganic materials to oligonucleotides and their self-assembled products has created hybrid nanostructures with a myriad of morphologies (e.g., micelles, nanorods, dendrimers, nanogels and liposomes), as well as their bulk gel products. In-depth characterization of these hybrids has revealed many factors (e.g., position of organic material/ligand, sequence length, number of bases) that influence the physicochemical properties of the hybrids. In revealing these factors, nanocarriers with improved stability, greater mechanical strength and uptake have been fabricated. Towards delivering therapeutic cargos, non-covalent strategies, such as the streptavidin-biotin interactions using protein conjugated oligonucleotides and electrostatic or intercalation interactions, which rely on drug binding to the anionic backbone or bases of DNA, respectively, have been leveraged with much success. These strategies have proven the potential of DNA hybrid systems to improve cargo stability in degrading environments. Consequently, better targeting, improved cellular uptake/internalization and drug delivery capacities have been demonstrated. The choice of material (i.e., organic or inorganic) for hybrid formation has been successful in addressing the drawbacks of pristine nanostructures and cargos, so their applications can be extended beyond cancer therapeutics. 

The success realized with nucleic acid hybrid systems provides a relevant basis to develop multifunctional antibacterial therapeutics that can address the eminent challenge of drug resistance. These nanocarriers can be leveraged to improve diagnostics of infectious diseases, restore sensitivity to antibiotics, deliver antibacterial molecules, as well as being designed to possess inherent antibacterial properties. In bacteria detection, the study by Setyawti et al. demonstrated internalization (both *E. coli* and *S. aureus*) of the tetrahedral hybrids, whereas Mela et al. showed surface binding with a circa 20% area coverage of bound origami nanostructures to the cell wall of *E. coli*, thereby acknowledging the importance of hybrid morphology/size. Nevertheless, binding studies were conducted within 15 minutes, whereas uptake was seen within 3 h. Spurred on by these advancements, we have the opportunity to improve bacteria detection to address the slow, imprecise and erroneous conventional approach currently used in detecting infections. In restoring bacteria sensitivity to antibiotics, ESBLs were targeted by the two reviewed studies, given their popularity as the main resistance mechanisms in enterobacteria [129,134]. While both approaches saw increased sensitivity to cephalosporins, greater potency is seen for the lipid-modified conjugates and their micellar nanostructures (~26×) compared to the tetrahedral system (~2×). While this can be centered on the potency of the antisense oligonucleotide itself, the rapid release/availability of the antisense sequence can also account for this observation, since it formed part of the rigid tetrahedral nanostructure. Conversely, the exposed oligonucleotide in the micellar formulation (forming the corona) is more readily available to its target following uptake. While other factors may account for this observation, it is rather obvious that the intracellular uptake and/or degradation of the nucleic acid nanostructures and their hybrid structures in bacterial pathogens are properly investigated, to give greater insight into designing these systems. In delivering antimicrobial cargos, intercalating antimicrobial drugs, cationic antimicrobial peptides and antibacterial lysozymes were reviewed. These studies demonstrated a direct correlation for bacteria uptake/binding or bacteria responsive release with bactericidal activity/efficacy. Using the tetrahedral nanostructures, greater potency was observed for actinomycin D, which was converse to the antimicrobial peptide (AMP) loaded gel system [127,136]. Between these two reports, we posit that this observation was due to the greater uptake and faster release of the bound drug from smaller nanostructures than the self-assembled hydrogel. For drug loading, because it is possible to achieve rapid drug entrapment at room temperature or at 4 °C, the loading strategies remain highly relevant for sensitive antimicrobial drugs. In the origami nanostructures, a synergistic slowing of growth was observed, potentially due to its binding to the bacteria. As one of the few hybrid origami nanostructures shown to exert antibacterial activity, the mechanisms surrounding the slowed growth of the unloaded hybrid need to be uncovered. Finally, in the vancomycin loaded hybrid system, drug loading was achieved during thermal hybridization without diminishing the activity of the antibiotic. This is advantageous, and demonstrates beyond post-loading strategies that are commonly used (after pre-assembling the nanostructures), that pre-loading strategies can also be employed without significantly compromising the activity of the drug. Additionally, we see first-hand the advantage of merging therapeutic DNA nanostructures and antimicrobial drugs to achieve other effects, such as synergistic anti-inflammatory activity.

Using nucleic acid self-assembled hybrid nanocarriers as antimicrobial therapeutics provides a real opportunity to develop multifunctional nanocarriers against systemic infections, topical/biofilm infections and intracellular infections. Being able to adapt nucleic acid delivery systems, their hybrids or mixed hybrid formulations will allow us simultaneously address pathogens that quickly cross membranes to persist as well as their planktonic forms with high efficiency. Moreover, incorporating responsiveness to external stimuli, like NIR, enzymes (e.g., nucleases, proteases, lipases) and environmental conditions within the infection site, is relevant to regulating drug release and the activity of therapeutic components in a specific manner. Additionally, there is room to expand coating strategies to be used on medical devices to prevent biofilm formation and simultaneously release therapeutic drugs against bacterial pathogens.

The application of these hybrid systems in antibacterial therapy is still in its infant stages, so there are several challenges that need to be addressed. While non-covalent interactions are attractive and have been used in many of the reviewed studies, they are challenged by:

- Non-specificity: encouraging specific interactions, such as aptamer-driven interactions with the hybrids, can be pursued.
- Rapid drug release: strategies that sustain the release of antimicrobial cargos, such as the use of liposomal vesicles and other coating methods, can be used.
- Stability concerns: towards clinical translation, future investigation of the antibacterial stability of the hybrids under conditions relevant to infections and translating these using in vivo conditions is important.
- Long-term storage: perceiving the integrity of the hybrids as solution or in their lyophilized form is a challenge, yet necessary to better understand the long-term storage of the hybrids.
- Complex formation: many antimicrobial agents are cationic/amphiphilic in nature, so complex formation may cause structural distortion of the nanomaterial. Co-delivery systems using multiple loading approaches can be pursued.
- Limited on loading higher concentrations of cationic cargos: high drug loading is challenged directly by the formation of complexes, which may significantly reduce the potency of the bound drug. Expanding to incorporate multiple antimicrobial agents or loading antimicrobial drugs into hybrid nanostructures with inherent antimicrobial activity provides room to achieve enhanced potency by addressing multiple targets.

To address some of these challenges, the incorporation of promising synthetic oligonucleotide analogues and mimics, such as locked nucleic acids (LNA), peptide nucleic acids (PNA), within DNA nanostructures can address stability concerns, improve specificity, enhance uptake, minimize immunogenicity and complex formation [40]. Adopting these analogues to fabricate DNA nanostructured hybrids is currently limited due to their high cost, which makes their use as standard oligonucleotides rather challenging [40]. Moreover, without incorporating functionality into these analogues, a very limited benefit to cost ratio in antimicrobial therapy can be achieved. Nevertheless, the optimization of new chemistries that simplify the synthesis in a time and cost-effective manner, generate greater yields and products of high purity are envisaged to address these challenges [143]. On the other hand, co-delivery systems also represent an exciting prospect for these hybrids. It has been demonstrated that nucleic acid nanostructures possess anti-inflammatory properties [144], which are highly relevant in several areas of infections such as topical wounds. Thus, further development of the hybrid systems should thoroughly investigate their role in modulating inflammation, as well as the effect of the various conjugated ligands on inflammation. Assuming similar anti-inflammatory activity as shown by pristine DNA nanostructures, there is room to synergistically enhance anti-inflammatory activity of drugs using lower concentrations (for toxic drugs) in combination with hybrid structures. Many infections are characterized by polymicrobial pathogens, thus investigating the efficacy of the hybrids using relevant models, and tailoring the observations to further improve the efficacy of the hybrids, will truly expand their in vivo capacity. Very few of the reviewed studies utilized in vivo models, so we envision that, by using in vivo infection models to assess the hybrids, greater insights into how the hybrids influence the microbiota and their efficacy at different sites (e.g., ophthalmic, wound, oral, systemic) can be outlined. Addressing these concerns to gain better understanding of the in vivo activity, bacteria internalization, degradation within infection sites and specificity, more efficient antibacterial platforms can be realized using nucleic acid hybrid platforms. 

In conclusion, translating DNA nanostructures from laboratory into clinics suffers from similar drawbacks, as seen with nanomedicine, which requires greater focus on the disease biology, rather than the means to reach the target. While the impact of the nanocarrier cannot be ignored, DNA nanocarriers intended for bacterial therapy should put greater focus on the infectious agent/disease irrespective of the drug cargo to further improve in vivo efficacy.

## Figures and Tables

**Table 1:** Summary of nucleic acid hybrid nanostructures prepared using organic and inorganic nanoparticles and their applications.

Group	Functionalization	Hybrid Nanostructure	Biological Application Tested	Representative References
**Organic**	Hydrophobic	Micelles & nanorods Micelles Liposome-like nanoparticle Tetrahedron & origami	NA Corneal infection Targeted cancer therapy Cancer therapy	[64] [57] [65] [66]
	Protein/peptides	Nanogels Hydrogel Hydrogel Tetrahedral cage	Cancer therapy Antidote (alcohol removal) Protein delivery NA	[67] [68] [69] [70]
**Inorganic**	Gold	Origami Dendrimer Tetrahedrons Nanosunflowers	NA NA Enzyme/cell delivery & bioimaging Gene therapy	[71] [72] [44] [73]
	Silver	Origami Origami Origami Nanoparticles	NA NA NA Cancer and antibacterial therapy	[74] [75] [76] [77]
	Quantum dot	Origami Hydrogel	NA Cancer therapy	[78] [79]

NA (Not applicable) means that the biological activity of the hybrid nanostructures was not tested.
